# Neglschisandrins C-D: Two New Dibenzocyclooctadiene Lignans from *Schisandra neglecta*

**DOI:** 10.3390/molecules13051148

**Published:** 2008-05-13

**Authors:** Min Chen, Zhihua Liao, Xiumei Xu, Yan Wen, Min Sun, Haoxiang Zhang, Wenhui Ma

**Affiliations:** 1 School of Pharmacy, Three Gorges Reservoir Region Key Laboratory of Eco-environmental Science (Ministry of Education), Southwest University, Chongqing 400715, P.R. China; 2 School of Life Sciences, Three Gorges Reservoir Region Key Laboratory of Eco-environmental Science (Ministry of Education), Southwest University, Chongqing 400715, P.R. China; E-mails: zhliao@swu.edu.cn (Liao); xxmei515@163.com (Xu); ff2@swu.edu.cn (Wen); jwcsm@swu.edu.cn (Sun); zhanghaoxing@yahoo.com.cn (Zhang); 3 School of Life Sciences, Linyi Normal University, Linyi, Shandong Province, 276005, P.R. China; 4 Department of Life Sciences, Hainan Normal University, Haikou, Hainan Province, 571158, P.R. China; E-mail: mawh08@126.com

**Keywords:** *Schisandra neglecta*, dibenzocyclooctadiene lignans, neglschisandrins C-D

## Abstract

Two new dibenzocyclooctadiene lignans, neglschisandrins C-D (**1**-**2**), were isolated from the stems of *Schisandra neglecta*. Their structures and stereochemistries were elucidated by spectroscopic methods, including 1D- and 2D-NMR and HR-ESI-MS techniques.

## Introduction

The stems or fruits of plants in the Schisandraceae family are widely used in China as tonics and astringent drugs for the treatment of rheumatic arthritis, traumatic injuries and related diseases [1]. Plants of the Schisandraceae are rich in lignans, especially dibenzocyclooctadiene ones, which have been found to possess some beneficial effects such as anti-HIV, antitumor, calcium antagonism and anti-lipid peroxidation properties, etc. [2,3,4,5,6]. In a previous study, two new dibenzocyclooctadiene lignans from the *Schisandra neglecta *were reported [7]. In our continuing efforts to identify bioactive natural products from the stems of *Schisandra* medicinal plants, a chemical investigation on the stems of *Schisandra* *neglecta* (Schisandraceae), indigenous to the Tibet Autonomous Region of China, was tested for inhibition of tumor cells growth and showed cytotoxic activity. Bioactivity-directed fractionation of this extract led to the isolation and identification of two new dibenzocyclooctadiene lignans, named neglschisandrins C-D (**1-2**). This paper deals with the isolation and characterization of these new compounds.

## Results and Discussion

**Figure 1:**
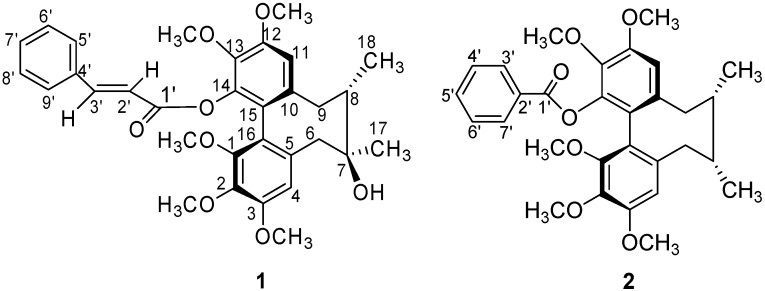
Structures of compounds **1-2**.

Repeated column chromatography of the Et_2_O-soluble fraction of the ethanol extract of the stems of *S. neglecta* yielded two new lignans **1** and **2 **(Figure 1), which have been named neglschisandrins C-D, respectively.

Neglschisandrin C (compounds **1**) was obtained as a colorless powder. A molecular formula of C_32_H_36_O_8_ was established by HR-ESI-MS (*m/z* 571.2302 [M+Na]^+^). The UV spectrum, with absorption maxima at 217, 252 and 280 nm, along with the ^1^H-NMR and ^13^C-NMR data mentioned below indicated that compound **1** was a dibenzocyclooctadiene lignan [8].

**Figure 2:**
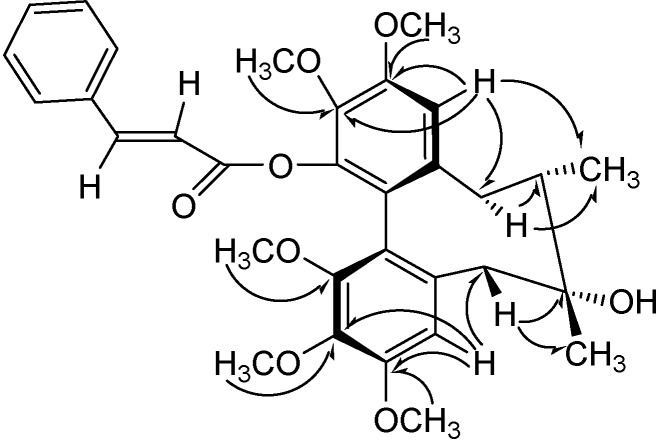
Key HMBC Correlations of compound **1.**

The ^1^H-NMR spectrum of **1** (Table 1) showed a singlet methyl signal (δ_H_ 1.28, 3H) and a doublet methyl signal (δ_H_ 0.88, *J*=7.2 Hz, 3H), indicating the presence of a tertiary methyl group attached to a carbon carrying a hydroxyl (δ_H_ 2.13, 1H, br s) and a secondary methyl group, which could be assigned to CH_3_-17 and CH_3_-18, respectively [9]. The presence of four methylene signals (*δ*_H_ 2.74, 1H, *d*, *J*=13.7 Hz; 2.37, 1H, *d*, *J*=13.7 Hz; 2.73, 1H, *dd*, *J*=14.3, 1.3 Hz and 2.44, 1H, *dd*, *J*=14.3, 7.7 Hz) indicated that, like the known heteroclitin H [10], compound **1** had no substitution at C-6 and C-9. Based on the HMQC spectrum, the protons at *δ*_H_ 2.74 and 2.37 were attached to the same carbon (*δ*_C_ 40.7), as were the protons at *δ*_H_ 2.73 and 2.44 (δ_C_ 34.4). Furthermore, HMBC correlations of *δ*_H_ 2.37 with *δ*_C_ 29.8 (C-17) and *δ*_C_ 71.9 (C-7) and of *δ*_H_ 2.73 with *δ*_C_ 15.9 (C-18) and *δ*_C_ 41.9 (C-8) indicated that *δ*_H_ 2.74 and 2.37 were H_2_-6 and that *δ*_H_ 2.73 and 2.44 were H_2_-9 (see Figure 2).

**Table 1:** NMR data of compounds **1**-**2** in CDCl3 (*δ* in ppm, *J* in Hz).

No	Compound 1		Compound 2	
	*δ*_C_	*δ*_H_ (*mult.*, *J* )	*δ*_C_	*δ*_H_ (*mult*., *J* )
1	151.8		151.2	
2	140.4		139.4	
3	152.5		152.9	
4	110.3	6.72(*s*)	107.4	6.44(*s*)
5	132.9		140.1	
6	40.7	6α: 2.74 (*d*, *J*=14.7) 6β: 2.37 (*d*, *J*=13.7)	35.4	2.36 (*dd*, *J*=13.4/9.7) 2.04 (*d*, *J*=13.3)
7	71.9		40.6	1.80 (*m*)
8	41.9	1.90 (*m*)	33.8	1.94 (*m*)
9	34.4	9α: 2.73 (*dd*, *J*=14.3/1.3) 9β: 2.44 (*dd*, *J*=14.3/7.7)	39.1	9α: 2.56 (*dd*, *J*=13.6/1.8) 9β: 2.66 (*dd*, *J*=13.6/7.4)
10	133.8		133.9	
11	113.0	6.56 (*s*)	113.1	6.74 ( *s*)
12	151.7		151.5	
13	139.8		139.7	
14	142.2		142.4	
15	123.1		123.5	
16	122.6		120.9	
17	29.8	1.28 (*s*)	21.6	1.00 (*d*, *J*=7.1)
18	15.9	0.88 (*d*, *J*=7.2)	12.9	0.81 (*d*, *J*=7.1)
7-OH	-	2.13 (*br s*)		
1-OMe	60.7	3.61 (*s*)	60.5	3.53 (*s*)
2-OMe	60.9	3.78 (*s*)	60.6	3.63 (*s*)
3-OMe	55.9	3.83 (*s*)	55.8	3.78 (*s*)
12-OMe	56.1	3.93 (*s*)	56.1	3.93 (*s*)
13-OMe	61.0	3.86 (*s*)	60.9	3.85 (*s*)
Cin: 1'	164.4	-		
2'	117.0	6.36 (*d*, *J*=16.0)		
3'	145.8	7.62 (*d*, *J*=16.0)		
4'	134.2	-		
5'/9'	128.1	7.44 (*m*)		
6'/8'	128.9	7.35 (*m*)		
7'	130.5	7.35 (*m*)		
Ben: 1'			164.1	
2'			129.8	
3'/7'			129.9	7.97 (*d*, *J*=7.3)
4'/6'			128.1	7.34 (*t*, *J*=7.8)
5'			132.9	7.49 (*t*, *J*=7.3)

The ^1^H-NMR spectrum of compound**1** also showed signals due to two aromatic protons (*δ*_H_ 6.72, 6.56, each *s*, 1H) and five methoxy group singlets (*δ*_H_ 3.61, 3.78, 3.83, 3.86 and 3.93, each 3H) on two aromatic rings. The ^1^H-NMR spectrum also showed the presence of a trans-cinnamic acid ester with proton signals at *δ*_H_ 6.36 and 7.62 (each 1H, *d*, *J*=16.0 Hz) and aromatic proton signals at *δ*_H_ 7.44 (2H, *m*) and 7.35 (3H, *m*). Carbon signals at *δ*_C_ 117.0, 145.8, 134.2, 128.1 (×2), 128.9 (×2) and 130.5, as well as carbonyl carbon at *δ*_C_ 164.4, supported this deduction [7].

HMBC correlations (Figure 2) of *δ*_H_ 6.56 with *δ*_C_ 40.7 (C-6) and *δ*_H_ 6.72 with *δ*_C_ 34.4 (C-9) suggested that these two protons were H-4 and H-11, respectively. Their corresponding carbon signals were assigned as *δ*_C_ 110.3 and 113.0, respectively, by HMQC techniques. Based on HMBC correlations of H-4 with the aromatic carbons at *δ*_C_ 140.4 and 152.5 and of H-11 with *δ*_C_ 139.8, 142.2 and 151.7, these five carbons were assigned to C-2, -3, -13, -14 and -12, respectively. The positions of the five methoxy substituents were elucidated from the HMBC cross peaks of *δ*_H_ 3.78, 3.61, 3.83, 3.86 and 3.93 with *δ*_C_ 140.4 (C-2), 151.8 (C-1), 152.5 (C-3), 139.8 (C-13) and 151.7 (C-12), respectively. Thus, the cinnamoxyl group should be located at C-14 position.

**Figure 3:**
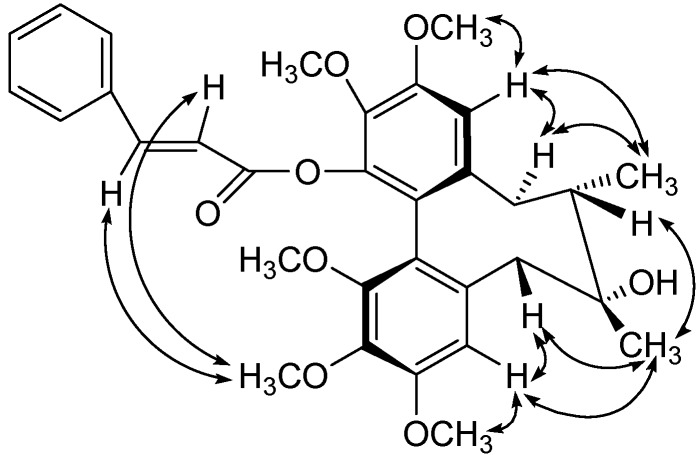
Key NOESY Correlations of compound **1.**

The circular dichroism (CD) spectrum showed a negative *Cotton* effect at 215 nm and a positive *Cotton* effect at 249 nm, indicating that compound **1** has a *R*-biphenyl configuration [11]. The NOESY cross peaks (see Figure 3) for H-11/CH_3_-18, H-11/H-9α, H-4/H-6β and H-9α/CH_3_-18 in compound **1** suggested a twist-boat-chair (TBC) conformation for the cyclooctadiene ring [12]. The stereochemical assignments in the cyclooctadiene ring of compound **1** were supported by other NOESY correlations of H-4/3-OMe, H-11/12-OMe, 2-OMe/H-2', 2-OMe/H-3', H-6β/CH_3_-17, H-4/CH_3_-17 and H-8/CH_3_-17. From the above data, the structure of compound **1** was elucidated as *(6R,7S,R-biar)-3-phenylacrylic acid-7-hydroxy-2,3,10,11,12-pentamethoxy-6,7-dimethyl-5,6,7,8-tetrahydrodibenzo[a,c]cycloocten-1-yl ester.*

Neglschisandrin D (**2**)**,** obtained as a colorless powder, had the molecular formula C_30_H_34_O_7_ according to HR-ESI-MS [*m/z *529.2180 ([*M*+Na]^+^)]. The UV absorptions (226, 250 and 279 nm) and NMR spectra (Table 1) indicated that compound **2** was also a dibenzocyclooctadiene-type lignan. Its IR, UV, CD and NMR spectra were similar to those of compound **1**, the differences between both compounds being the substituents at C-14 and the C-7. 

**Figure 4:**
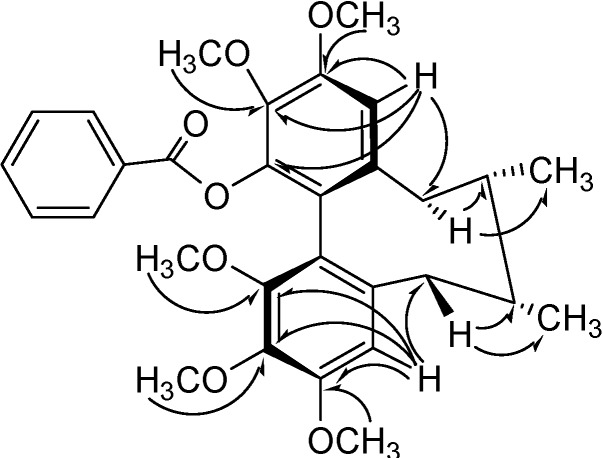
Key HMBC and NOESY Correlations of compound **2.**

In the cyclooctadiene ring, two *doublet* methyl signals (δ1.00, 0.81, each 3H, *J*=7.1Hz) were assigned to 7-Me and 8-Me, respectively. This suggested that there was no substitution at C-7 and C-8, and these two methyl groups were in *cis*-orientation [13]. Comparing the NMR spectrum of compound **2** with that of compound **1** (Table 1), the aromatic proton signals at *δ*_H_ 7.97 (2H, *d, J*=7.3Hz), 7.34 (2H, *t, J*=7.8 Hz) and 7.49 (1H, *t, J*=7.3 Hz) and carbon signals at *δ*_C_ 129.8, 129.9(x2), 128.1(x2) and 132.9, as well as carbonyl carbon at *δ*_C_ 164.1, showed that the cinnamoyl group in compound **1** was replaced by an benzoyl group in compound **2** (see Figure 4).

**Figure 5:**
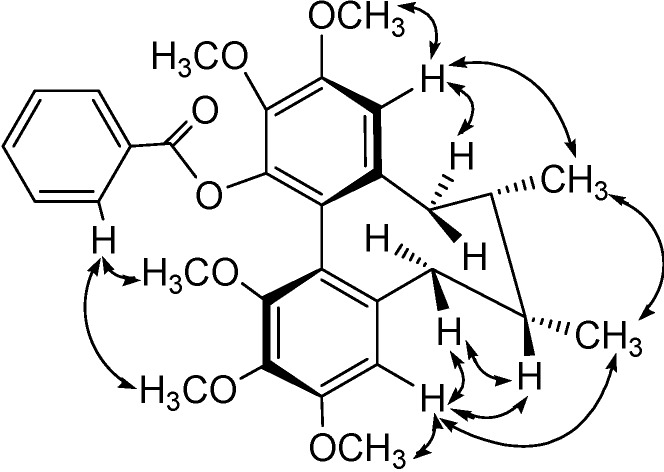
Key NOESY Correlations of **2**.

The circular dichroism (CD) spectrum showed a negative *Cotton* effect at 210 nm and a positive one at 247 nm, indicating that compound **1** has a *R*-biphenyl configuration. The NOESY cross peaks (Figure 5) for H-4 with CH_3_-17, H-4/H-6β, H-11 with H-9α and H-11 with CH_3_-18 in compound **2** suggested a twist-boat-chair (TBC) conformation for the cyclooctadiene ring. The stereochemical assignments in the cyclooctadiene ring of **1** were supported by other NOESY correlations of H-4/3-OMe, H-11/12-OMe, H-6β/H-7, H-4/H-7, CH_3_-17/CH_3_-18, H-7'/1-OMe and H-7'/2-OMe (see Figure 4). From the above data, the structure of compound **2** was elucidated as *(6S,7S,R-biar)benzoic acid 2,3,10,11,12-pentamethoxy-6,7-methyl-5,6,7,8-tetrahydrodibenzo[a,c]cycloocten-1- yl ester*.

## Experimental

### General

TLC: Silica-gel plates GF_254_ (Yan-tai Institute of Chemical Technology). Column chromatography (CC): Silica gel (200-300 mesh or 300-400 mesh: Qingdao Marine Chemical Factory). Prep HPLC: Amersham UV-900, with RP-C18 column (250×10 mm). UV [in anh. MeOH; λ_max_ in nm (log ε)]: Hitachi U-3010 spectrophotometer. CD Spectra [λ in nm (∆ε in mdeg)]: Jasco-810 spectropolarimeter. Optical rotation (ORD): JASCO P-1020 spectropolarimeter. IR Spectra (KBr pellets; in cm^-1^): Avatar 360-ESP spectrophotometer (Thermo Nicolet). ^1^H- (400 MHz) and ^13^C-NMR (100 MHz) spectra in CDCl_3_ soln.; *δ* in ppm rel. to Me_4_Si, *J* in Hz): Bruker DRX400 Spectrometer. ESI-MS (*m/z*): Bio TOF Q spectrometer; HR-ESI-MS (*m/z*): Bruker Dalonics-BioToF Q spectrometer.

### Plant Material

Stems of *Schisandra neglecta* were collected in Lin-zhi County, Xi-zang Autonomous Region, People’s Republic of China, in September of 2004, and identified by Associate Professor Hong-ping Deng of the School of Life Sciences, SouthWest University. A voucher specimen (MC-LZ-040901) is deposited in the Herbarium of Medicinal Plant, School of Life Sciences, SouthWest University, Chongqing, People’s Republic of China.

### Extraction and Isolation

Air-dried stems of *Schisandra neglecta* (5 kg) were ground and extracted exhaustively with 95% ethanol at room temperature. The EtOH extract was evaporated *in vacuo* to yield a semisolid (430 g), which was suspended in H_2_O (1 L) and extracted with Et_2_O (5×1L). This ether solution was concentrated to yield 112 g of residue, which was subjected to CC [SiO_2_, 1.5 kg, petroleum ether (PE)/acetone gradient]. Fraction 4 (eluted with PE/acetone 9:1) was subjected to repeated CC (eluted with PE/EtOAc 15:1) and prep*.* RP-HPLC (MeOH/H_2_O 70:30) to yield compound **2** (2 mg). Fraction 5 (eluted with PE/acetone 8:2) was subjected to repeated CC (eluted with PE/CHCl_3_ 1:1) and prep*.* RP-HPLC (MeOH/H_2_O 80:20) to give compound **1** (29 mg).

*(6R,7S,R-biar)-3-phenylacrylic acid-7-hydroxy-2,3,10,11,12-pentamethoxy-6,7-dimethyl-5,6,7,8-tetra-hydrodibenzo[a,c]cycloocten-1-yl ester* (*neglschisandrin C*, **1**). Colorless powder; UV: 217 (4.44), 252 (4.05), 280 (3.23); CD (*c*=0.08, MeOH): nm (Δε) 249 (+39.76), 234 (+55.71), 215 (-76.79); 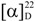
=+72.1°(*c*=0.48, MeOH); IR: 3415, 1726, 1636, 1594, 1494; ^ 1^H-NMR and ^13^C-NMR: see Table 1; ESI-MS *m/z*: 571.1 ([*M*+Na]^+^); HR-ESI-MS: *found* 571.2290 ([*M*+Na]^+^, C_32_H_36_O_8_Na, *calc.* 571.2302).

*(6S,7S,R-biar)-benzoic acid-2,3,10,11,12-pentamethoxy-6,7-methyl-5,6,7,8-tetrahydrodibenzo[a,c]-cycloocten-1-yl ester (neglschisandrin D,* **2**). Colorless powder; UV: 226 (4.42), 250 (4.12), 279 (3.71); CD (*c*=0.4, MeOH): nm (Δε) 247 (+312.52), 233 (+389.88), 211 (-278.28); 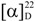
=+79.6°(*c*=1.30, MeOH); IR: 1738, 1597, 1493, 707; ^1^H-NMR and ^13^C-NMR: see Table 1; ESI-MS *m/z*: 529.5 ([*M*+Na]^+^); HR-ESI-MS: *found* 529.2180 ([*M*+Na]^+^, C_30_H_34_O_7_Na, *calc. *529.2197).

## References

[1] (1977). Dictionary of Chinese Traditional Medicines.

[2] Chen M., Kilgore N., Lee K. H., Chen D. F. (2006). Rubrisandrins A and B, Lignans and Related Anti-HIV Compounds from *Schisandra rubriflora*. J. Nat. Prod..

[3] Chen D. F., Zhang S. X, Kozuka M., Sun Q. Z., Feng J., Wang Q., Mukainaka T., Nobukuni Y., Tokuda H., Nishino H., Wang H. K., Morris-Natschke S. L., Lee K. H. (2002). Interiotherins C and D, two new lignans from *Kadsura interior* and antitumour-promoting effects of related neolignans on Epstein-Barr virus activation. J. Nat. Prod..

[4] Chen D. F., Zhang S. X., Xie L., Xie J. X., Chen K., Kashiwada Y. B., Zhou N., Wang P., Cosentino L. M., Lee K. H. (1997). Structure-Activity Corelations of Gomisin G-Related Anti-HIV Lignans from *Kadsura interior* and of Related Synthetic Analogues. Bioorg. Med. Chem..

[5] Zhang X. M, Chen D. F., He X. J., Yang S., Zheng P., Jiang M. H. (2000). The Blocking Effects of Heteroclitin D and Gomisin J on L-type Calcium Channels in Ventricular Cells of Guinea Pig. Acta Pharmacol. Sin..

[6] Peng H. L., Chen D. F., Lan H. X., Zhang X. M., Gu Z., Jiang M. H. (1996). Anti-Lipid Peroxidation of Gomisin J on Liver Mitochodria and Cultured Myocardial Cells. Acta Pharmacol. Sin..

[7] Chen M., Xu X. M., Liao Z. H., Dong L., Li L, Huang C. Z. (2008). Neglschisandrins A-B: Two New Dibenzocyclooctadiene Lignans from *Schisandra neglecta*. Molecules.

[8] Jia Z. W., Liao Z. X., Chen D. F. (2005). Two new dibenzocyclooctadiene lignans from the water extract of *Kadsura spp*. Helv. Chim. Acta.

[9] Chen M., Liao Z. X., Chen D. F. (2004). Four New Dibenzocyclooctadiene Lignans from *Kadsura renchangiana*. Helv. Chim. Acta.

[10] Chen M., Jia Z. W., Chen D. F. (2006). Heteroclitin H, a New Lignans from *Kadsura heteroclita*. J. Asian Nat. Prod. Res..

[11] Li H. R., Feng Y. L., Yang Z. G., Wang  J., Daikonya A., Kitanaka S., Xu L. Z., Yang S. L. (2006). New lignans from* Kadsura coccinea* and their nitric oxide inhibitory activities. Chem. Pharm. Bull..

[12] Liu J.S., Li L. (1993). Schisandtherins L-O and acetylschisantherin L from *Kadsra coccinea*. Phytochemistry.

[13] Ghera E., Ben-David Y., Becker D. (1977). Desoxyschizandrin, stereochemistry and total synthesis. Tetrahedron Lett..

